# Puerperal Eclampsia

**Published:** 1896-09

**Authors:** T. M. Yett


### TEXAS MEDICAL JOURNAL,

ESTABLISHED JULY, 1885.

PUBLISHED MONTHLY.—SUBSCRIPTION $2.00 A YEAR.

VOL. XII. AUSTIN, SEPTEMBER, 1896. NO. 3

### Original Contributions.

For the Texas Medical Journal.

### PUERPERAL ECLAMPSIA.

BY T. M. YETT, M. D.

[Read at Austin District Medical Society, June 21, 1896.]

THE treatment of puerperal eclampsia has in former years baffled the skill of the practitioner. Statistics show a mortality of 35 per cent. in cases treated by bleeding, and 11 per cent. where anesthetics were in use.

A mortality so large as this must necessarily awaken in the mind an earnest desire for methods of treatment more affectual than these, and there can be little doubt that if the death rate from this cause is in the future to be materially reduced, it must be by a careful and earnest investigation, and an observation dictated by the same spirit of clinical facts: and any new idea I shall gather from your discussions of this subject to-day will better prepare me to treat this formidable disease.

I shall not enter upon the pathology and etiology of this alarming disorder.

That albuminuria and puerperal eclampsia are of simultaneous occurrence, or mutually dependent upon each other, in the vast majority of cases, is an assertion not likely, in modern times, to be seriously controverted.

What may be called the obstetrical treatment of eclampsia involves a more particular reference to the stage at which the convulsion occurs. The rules laid down in our text-books as to what is best to be done when the convulsion occurs before or after delivery, with any complications that may arise, are well understood and need not be mentioned in this paper. And I shall not take up your valuable time by mentioning these older methods, which every physician has had indelibly impressed upon his mind before he assumed the duties of obstetrician.

While the older remedies are still being used by a great many, yet others are entering new fields of investigation, and we find the intravenous injection of saline solution, the hypodermic injection of pilocarpine and others, which all aim at the elimination of the morbid material from the system by the emunctories that has accumulated in the blood during the period of pregnancy.

But the remedy I wish to speak of particularly to-day is the hypodermic injection of veratrum veride; and while we should not pin our faith to any remedy and say it will absolutely cure all cases, yet I believe it is as near a specific as any remedy can be for a particular disease—as much so as quinine is for malaria. More than twenty years ago, when Dr. Fearn advocated heroic doses of veratrum veride in puerperal eclampsia, the profession thought him too bold and reckless in the use of a remedy so depressing in its effects. But they have been giving it a larger trial each year, and more recently some clinicians have given additional evidence of the great results obtained by this treatment. Some of the great obstetric teachers of the day are recommending it to their classes. Herst, Lusk and Parvin mention the treatment and state its advantages. And while the doses used seem to be large to us, who are accustomed to get marked circulatory sedation in the early stages of acute inflammatory troubles with much smaller doses, yet the stomach and bowels seem to act as safety valves, hence very few deaths have resulted from these heroic doses. In reality the boundary line of danger is not passed, for we all know that we are to be governed by the effect and not the size of dose. Now we are led to ask, what is the modus operandi by which we get such good results? In the first place it acts as a cathartic and thus unloads the alimentary canal, which is very essential. Second, it produces copious perspiration, which is another means of relieving the blood of impurities. Third, it relaxes the spasm of the renal vessels, causing marked increase in the flow of urine, thereby not only relieving the congestion of the kidneys but relaxing the peripheral blood vessels. Fourth, acting as a powerful depresser of the circulation, it is said to "bleed the woman into her own vessels," relieving congestion of the brain. Fifth, it acts as a sedative to the motor tracts of the spinal cord and quiets nervous excitation. Many times the patient does not recover consciousness between the paroxysms, and it may be almost impossible to get her to swallow, but by the hypodermic use of ver. veride, we are relieved of this embarrassment.

Was called hurriedly on the night of June 6th, 1894, six miles out of town to see Mrs. D., "messenger saying she was having fits." When I arrived, found large and plethoric woman about eighteen years of age; first confinement; had been delivered of a large child about 4 p. m. by a mid-wife. I found her in an unconscious condition, had had seven or eight convulsions, and one came on while I was trying to get a dose of chloral in her mouth. Pulse, 120; temperature, about normal. As soon as spasm was off, gave her ten drops Norwood's tr. ver. veri. hypodermically; was threatened with another convulsion, but it passed off, and in one hour her pulse was 100. Gave another dose of eight drops; pulse went down to 60; by morning she was conscious, and had no return of convulsions.

